# Supplementary material for: Influence of adiposity and physical activity on the cardiometabolic association pattern of lipoprotein subclasses to aerobic fitness in prepubertal children
Source: PLoS One. 2021 Nov 18;16(11):e0259901. doi: 10.1371/journal.pone.0259901 (PMC8601570; doi:10.1371/journal.pone.0259901)
Supplement: S6 Table — (DOCX) [file pone.0259901.s006.docx]

**S6 Table. Mean and standard deviation for demography, anthropometry and metabolic health for the children**.

|  | **Overall (n = 841)** | **Boys (n = 424)** | **Girls (n = 417)** |
| --- | --- | --- | --- |
| **Demography** |  |  |  |
| Age (years) | 10.2 (0.3) | 10.2 (0.3) | 10.2 (0.3) |
| **Anthropometry** |  |  |  |
| Body mass (kg) | 37.0 (8.1) | 36.8 (7.8) | 37.2 (8.3) |
| Height (cm) | 142.9 (6.7) | 143.1 (6.7) | 142.6 (6.8) |
| BMI (kg/m^2^) | 18.0 (3.0) | 17.9 (2.9) | 18.1 (3.1) |
| Overweight and obese (%) | 20.8 | 20.0 | 21.5 |
| Waist circumference (cm) | 61.9 (7.5) | 62.2 (7.3) | 61.6 (7.7) |
| Waist:height (ratio) | 0.43 (0.05) | 0.43 (0.05) | 0.43 (0.05) |
| **Indices of metabolic health** |  |  |  |
| Andersen test (m) | 898 (103) | 925 (112) | 871 (85) |
| Systolic blood pressure (mmHg) | 105.2 (8.4) | 105.3 (8.2) | 105.2 (8.6) |
| Diastolic blood pressure (mmHg) | 57.7 (6.2) | 57.4 (6.0) | 58.1 (6.3) |
| Total cholesterol (mmol/l) | 4.46 (0.69) | 4.46 (0.70) | 4.46 (0.68) |
| LDL-cholesterol (mmol/l) | 2.51 (0.64) | 2.50 (0.65) | 2.53 (0.62) |
| HDL-cholesterol (mmol/l) | 1.59 (0.35) | 1.63 (0.34) | 1.55 (0.35) |
| Total:HDL-cholesterol (ratio) | 2.91 (0.71) | 2.82 (0.66) | 2.99 (0.74) |
| Triglyceride (mmol/l) | 0.78 (0.38) | 0.72 (0.31) | 0.84 (0.42) |
| Glucose (mmol/l) | 4.98 (0.32) | 5.02 (0.31) | 4.94 (0.33) |
| Insulin (pmol/l) | 55.0 (29.8) | 48.9 (24.1) | 61.1 (33.6) |
| HOMA (index) | 1.71 (0.98) | 1.54 (0.83) | 1.89 (1.09) |

BMI = body mass index; LDL = low density lipoprotein; HDL = high density lipoprotein; HOMA = homeostasis model assessment.

PA measurement

PA was measured using the ActiGraph GT3X+ accelerometer (Pensacola, FL, USA) ^23^. Participants were instructed to wear the accelerometer at the waist at all times over seven consecutive days, except during water activities (swimming, showering) or while sleeping. Units were initialized at a sampling rate of 30 Hz. Data for the vertical axis were analyzed at 1-second epochs using the KineSoft analytical software version 3.3.80 (KineSoft, Loughborough, UK). Data were restricted to hours 06:00 to 23:59. In all analyses, consecutive periods of ≥ 60 minutes of zero counts were defined as non-wear time ^24^. We applied wear time requirements of ≥ 8 hours/day and ≥ 4 days/week to constitute a valid measurement.

We created 23 PA variables of total time (min/day) to capture movement in narrow intensity intervals throughout the spectrum, from 0–99 to ≥ 10000 cpm.

Usikker på om dette er med?

For the purpose of reporting descriptive statistics, we used the Evenson cut points of 0–99, 100–2295, 2296–4011, ≥ 4012, and ≥ 2296 cpm for SED, LPA, MPA, VPA, and MVPA ^25 26^, respectively. We also reported achievement of the guideline PA level (mean of ≥ 60 min MVPA/day).

refs

23. John D, Freedson P. ActiGraph and Actical physical activity monitors: a peek under the hood. *Med Sci Sports Exerc* 2012;44(1 Suppl 1):S86-S89.

24. Aadland E, Andersen LB, Anderssen SA, et al. A comparison of 10 accelerometer non-wear time criteria and logbooks in children. *BMC Public Health* 2018;18:9. doi: 10.1186/s12889-018-5212-4

25. Evenson KR, Catellier DJ, Gill K, et al. Calibration of two objective measures of physical activity for children. *J Sports Sci* 2008;26(14):1557-65. doi: 10.1080/02640410802334196

26. Trost SG, Loprinzi PD, Moore R, et al. comparison of accelerometer cut points for predicting activity intensity in youth. *Med Sci Sports Exerc* 2011;43(7):1360-68. doi: 10.1249/MSS.0b013e318206476e
