# Supplementary material for: Influence of adiposity and physical activity on the cardiometabolic association pattern of lipoprotein subclasses to aerobic fitness in prepubertal children
Source: PLoS One. 2021 Nov 18;16(11):e0259901. doi: 10.1371/journal.pone.0259901 (PMC8601570; doi:10.1371/journal.pone.0259901)
Supplement: S7 Table — (DOCX) [file pone.0259901.s007.docx]

**S7 Table.** **Time spent in different PA intensity intervals (mean (SD)) with epoch setting 1s**.

| **Physical activity intensity (cpm)** | **Minutes** |
| --- | --- |
| **0–99** | 597 (57) |
| **100–249** | 18 (3) |
| **250–499** | 21 (4) |
| **500–999** | 30 (6) |
| **1000–1499** | 23 (5) |
| **1500–1999** | 20 (4) |
| **2000–2499** | 15 (3) |
| **2500–2999** | 12 (3) |
| **3000–3499** | 11 (3) |
| **3500–3999** | 7.9 (2.4) |
| **4000–4499** | 6.4 (2.1) |
| **4500–4999** | 5.7 (2.0) |
| **5000–5499** | 4.0 (1.4) |
| **5500–5999** | 3.3 (1.2) |
| **6000–6499** | 3.0 (1.1) |
| **6500–6999** | 2.2 (0.7) |
| **7000–7499** | 1.8 (0.7) |
| **7500–7999** | 1.6 (0.7) |
| **8000–8499** | 1.1 (0.5) |
| **8500–8999** | 1.3 (0.4) |
| **9000–9499** | 0.9 (0.4) |
| **9500–9999** | 0.6 (0.3) |
| **≥ 10000** | 7.6 (6.5) |
