# Supplementary material for: Influence of adiposity and physical activity on the cardiometabolic association pattern of lipoprotein subclasses to aerobic fitness in prepubertal children
Source: PLoS One. 2021 Nov 18;16(11):e0259901. doi: 10.1371/journal.pone.0259901 (PMC8601570; doi:10.1371/journal.pone.0259901)
Supplement: S1 File — Mean and standard deviation of lipoprotein features and their correlations with aerobic fitness (AF), physical activity (PA) and adiposity measures. (DOCX) [file pone.0259901.s009.docx]

**Mean and standard deviations of lipoprotein features and their correlations with aerobic fitness (AF), physical activity (PA) and adiposity measures**.

Mean and standard deviation for concentrations of lipoprotein classes (mmol/L):

TC (4.35 ± 0.66), TG (0.74 ± 0.38), CM (0.036 ± 0.051), VLDL (1.07 ± 0.51), LDL (2.42 ± 0.50), HDL (1.57 ± 0.25), CM-1 (0.021 ± 0.031), CM-2 (0.014 ± 0.020), VLDL-L1 (0.059 ± 0.064), VLDL-L2 (0.14 ± 0.14), VLDL-L3 (0.36 ± 0.18), VLDL-M (0.26 ± 0.11), VLDL-S (0.25 ± 0.06), LDL-L (0.79 ± 0.17), LDL-M (1.04 ± 0.22), LDL-S 0.42( ± 0.09), LDL-VS (0.18 ±0.04 ), HDL-VL1 (0.020 ± 0.004), HDL-VL2 (0.067 ± 0.031), HDL-L (0.36 ± 0.16), HDL-M (0.56 ± 0.08), HDL-S (0.40 ± 0.04), and, HDL-VS (0.16 ± 0.01).

Average particle size (nm) for the main lipoprotein classes: VLDL (42.5 ± 2.8), LDL (25.77 ± 0.13) and, HDL (10.87 ± 0.21).

Correlations of AF, lipoproteins, PA and adiposity.

Correlation coefficients are tabulated in S7 Table. It shows that AF has negative correlations in the range -0.2 to -0.25 with CM, VLDL and LDL and all their subclasses except VLDL-S (which is often labelled as intermediate density lipoprotein, IDL) and LDL-L, and the average size of VLDL particles. HDL, HDL-VL2, HDL-L, and average size of HDL particles all have a positive correlation above 0.2 to AF. S1 reveals that the correlation of AF is stronger to adiposity than to the lipoproteins, i.e., -0.43 to BMI, -0.46 to WC/H, and -0.51 for skinfold. Correlation of AF to PA increases with the intensity of PA and reaches a flat maximum of -0.43 at 7500 counts/min. The correlation patterns of the adiposity measures to lipoproteins are qualitatively the same as for AF, but opposite and stronger. Thus, correlations are between -0.36 and -0.43 for HDL, the HDL subclasses of very large and large particles, and the average size of HDL particles. The correlation pattern between adiposity and PA are almost opposite with that of AF to PA, but the correlations are slightly smaller. For the lipoproteins and PA, correlations are weak at low intensities but gradually increase with intensity and around 7500 counts/min the correlation pattern coincides with the correlation pattern of PA to AF.
