# Supplementary material for: Influence of adiposity and physical activity on the cardiometabolic association pattern of lipoprotein subclasses to aerobic fitness in prepubertal children
Source: PLoS One. 2021 Nov 18;16(11):e0259901. doi: 10.1371/journal.pone.0259901 (PMC8601570; doi:10.1371/journal.pone.0259901)
Supplement: Attachment — Submitted filename: renamed_7e70c.docx [file pone.0259901.s010.docx]

**COMMENTS – Manuscript PONE-D-21-18590**

Title: “Influence of adiposity and physical activity on the cardiometabolic association pattern of lipoprotein subclasses to aerobic fitness in prepubertal children”

**Important note: This review pertains only to** **‘statistical aspects’ of the study and so** **‘clinical aspects’ [like medical importance,** **relevance of the study,** **‘clinical significance and implication(s)’ of the whole study, etc.] are to be evaluated [should be assessed] separately/independently. Further please note that any ‘statistical review’ is generally done under the assumption that (such) study specific methodological [as well as execution] issues are perfectly taken care of by the investigator(s). This review is not an exception to that and so does not cover clinical aspects {however, seldom comments are made only if those issues are intimately / scientifically related & intermingle with ‘statistical aspects’ of the study}. Agreed that ‘statistical methods’ are used as just tools here, however, they are vital part of methodology [and so should be given due importance].**

**COMMENTS:** Your ABSTRACT is well drafted but assay type. Please note that it is preferable to divide the ABSTRACT with small sections like ‘Objective(s)’, ‘Methods’, ‘Results’, ‘Conclusions’, etc. which is an accepted practice of most of the good/standard journals [including this one]. It will definitely be more informative then, I guess, whatever the article type may be.

“Why [refer to ‘Availability of data’ section on page 14] the data analyzed in this article are added as Suppl. Mat.”? Moreover, note that most of the ‘Correlations of AF, lipoproteins, physical activity and adiposity’ as reported/displayed in Suppl. Mat. 2 are generally ‘low’. In this context, please read the following [which is pasted from one standard textbook on ‘Research Methodology’]. I am sure that the authors already know these things, however, it is very essential to keep the limitations in mind while interpreting results {which unfortunately are not interpretated adequately}.

Statistical test usually used to assess significance of Pearson’s ‘Correlation coefficient (r)’ is ‘t’ [where t = { r √ [(n-2) / (1-r^2^)] }for df=n-2, n is sample size] and here Ho is that the population/standard value of ‘r’ is zero. You need r=0.878 to be significant at 5% when n=5 but you need r=0.273 only, if n=50 & you need r=0.088 only, if n=500. Because ‘P-value’ heavily depends on sample size, it is customary to use the (available in most text books on ‘Biostatistics’ or on ‘www/net’) guidelines [very strongly suggesting] to consider an absolute value of ‘Correlation coefficient’ for interpreting positive or negative correlations (and do not rely only on corresponding ‘P’-value but also consider an absolute value of ‘Correlation coefficient’). [This argument is equally applicable to non-parametric Spearman’s ‘Correlation coefficient (ρ)’ as well.]

I request the authors to confirm the level of measurement of “Aerobic fitness test” scores [as said in concerned section that the test measured the total distance covered during a 10-minutes run. {and that Children ran from one end-line to another (20 m apart) in a to-and-fro movement intermittently, with 15-second work periods and 15-second breaks}. Moreover, you stated that “We used a PA descriptor of 23 intervals defined by counts per minute (cpm) covering the entire intensity spectrum”. In this context (just reminder), please read the following [which is again pasted from the same textbook on ‘Research Methodology’].

Though the measures/tools used are appropriate, most of them yield data that are in [at the most] ‘ordinal’ level of measurement [and not in ratio level of measurement for sure {as the score two times higher does not indicate presence of that parameter/phenomenon as double (for example, a Visual Analogue Scales VAS score or say ‘depression’ score)}]. Then application of suitable non-parametric test(s) is/are indicated/advisable [even if distribution may be ‘Gaussian’ (i.e. normal)]. Agreed that there is/are no non-parametric test(s)/technique(s) available to be used as alternative in all situation(s) [suitable / most desired/applicable], but should be used whenever/wherever they are available.

It is true that variable ‘time’ is ‘ratio’ but the procedure to record it could change level of measurement. One should just watch-out though it is definitely appreciable that “All variables were thus log-transformed, mean-centered and standardized to unit variance prior to the statistical analysis. After log transformation, normal probability plots showed that only CM, VLDL and a few of their subclasses in addition to TG still deviated from normal distribution”.

Further I request the authors to read the article {just for information, authors are likely to have done this already} titled ‘Relative Importance of Borderline and Elevated Levels of Coronary Heart Disease Risk Factors’ (Ann Intern Med. 2005;142:393-402) & kindly note that I am not asking you to change the study design, but frankly speaking, ‘clinical aspects’ [like medical importance, relevance of the study, ‘clinical significance and **implication(s)** of the whole study, etc.] are not very clear and so in my considered opinion, should be assessed separately/independently.
