# Supplementary material for: Influence of adiposity and physical activity on the cardiometabolic association pattern of lipoprotein subclasses to aerobic fitness in prepubertal children
Source: PLoS One. 2021 Nov 18;16(11):e0259901. doi: 10.1371/journal.pone.0259901 (PMC8601570; doi:10.1371/journal.pone.0259901)
Supplement: Attachment — Submitted filename: Response to reviewers.docx [file pone.0259901.s011.docx]

**Response to reviewers**

PONE-D-21-18590

Influence of adiposity and physical activity on the cardiometabolic association pattern of lipoprotein subclasses to aerobic fitness in prepubertal children

PLOS ONE

Dear Dr. Kvalheim,

Thank you for submitting your manuscript to PLOS ONE. After careful consideration, we feel that it has merit but does not fully meet PLOS ONE’s publication criteria as it currently stands. Therefore, we invite you to submit a revised version of the manuscript that addresses the points raised during the review process.

Please submit your revised manuscript by Oct 31 2021 11:59PM. If you will need more time than this to complete your revisions, please reply to this message or contact the journal office at [plosone@plos.org](mailto:plosone@plos.org). When you're ready to submit your revision, log on to https://www.editorialmanager.com/pone/ and select the 'Submissions Needing Revision' folder to locate your manuscript file.

Please include the following items when submitting your revised manuscript:

- A rebuttal letter that responds to each point raised by the academic editor and reviewer(s). You should upload this letter as a separate file labeled 'Response to Reviewers'.
- A marked-up copy of your manuscript that highlights changes made to the original version. You should upload this as a separate file labeled 'Revised Manuscript with Track Changes'.
- An unmarked version of your revised paper without tracked changes. You should upload this as a separate file labeled 'Manuscript'.

If you would like to make changes to your financial disclosure, please include your updated statement in your cover letter. Guidelines for resubmitting your figure files are available below the reviewer comments at the end of this letter.

If applicable, we recommend that you deposit your laboratory protocols in protocols.io to enhance the reproducibility of your results. Protocols.io assigns your protocol its own identifier (DOI) so that it can be cited independently in the future. For instructions see: <https://journals.plos.org/plosone/s/submission-guidelines#loc-laboratory-protocols>. Additionally, PLOS ONE offers an option for publishing peer-reviewed Lab Protocol articles, which describe protocols hosted on protocols.io. Read more information on sharing protocols at <https://plos.org/protocols?utm_medium=editorial-email&utm_source=authorletters&utm_campaign=protocols>.

We look forward to receiving your revised manuscript.

Kind regards,

Martin Senechal, PhD
Academic Editor
PLOS ONE

Journal requirements:

When submitting your revision, we need you to address these additional requirements.

1. Please ensure that your manuscript meets PLOS ONE's style requirements, including those for file naming. The PLOS ONE style templates can be found at

https://journals.plos.org/plosone/s/file?id=wjVg/PLOSOne_formatting_sample_main_body.pdf and

<https://journals.plos.org/plosone/s/file?id=ba62/PLOSOne_formatting_sample_title_authors_affiliations.pdf>.

2. Thank you for stating the following in the Acknowledgments Section of your manuscript:

“The study was funded by the Research Council of Norway (grant number 221047/F40) and the Gjensidige Foundation (grant number 1042294). None of the funding agencies had any role in the study design, data collection, analysis or interpretation of the data, or in writing the manuscripts.”

We note that you have provided funding information that is not currently declared in your Funding Statement. However, funding information should not appear in the Acknowledgments section or other areas of your manuscript. We will only publish funding information present in the Funding Statement section of the online submission form.

Please remove any funding-related text from the manuscript and let us know how you would like to update your Funding Statement. Currently, your Funding Statement reads as follows:

“SAA, grant number 221047/F40, The Research Council of Norway, https://www.forskningsradet.no/, and GKR, grant number 1042294, The Gjensidige Foundation, https://www.gjensidigestiftelsen.no/, none of the funders played any role in the study design, data collection and analysis, decision to publish, or preparation of the manuscript.”

Please include your amended statements within your cover letter; we will change the online submission form on your behalf.

3. Your ethics statement should only appear in the Methods section of your manuscript. If your ethics statement is written in any section besides the Methods, please delete it from any other section.

Additional Editor Comments (if provided):


[Note: HTML markup is below. Please do not edit.]

Reviewers' comments:

Reviewer's Responses to Questions

**Comments to the Author**

1. Is the manuscript technically sound, and do the data support the conclusions?

The manuscript must describe a technically sound piece of scientific research with data that supports the conclusions. Experiments must have been conducted rigorously, with appropriate controls, replication, and sample sizes. The conclusions must be drawn appropriately based on the data presented.

Reviewer #1: Partly

Reviewer #2: Partly

2. Has the statistical analysis been performed appropriately and rigorously?

Reviewer #1: I Don't Know

Reviewer #2: Yes

3. Have the authors made all data underlying the findings in their manuscript fully available?

The [PLOS Data policy](http://www.plosone.org/static/policies.action#sharing) requires authors to make all data underlying the findings described in their manuscript fully available without restriction, with rare exception (please refer to the Data Availability Statement in the manuscript PDF file). The data should be provided as part of the manuscript or its supporting information, or deposited to a public repository. For example, in addition to summary statistics, the data points behind means, medians and variance measures should be available. If there are restrictions on publicly sharing data—e.g. participant privacy or use of data from a third party—those must be specified.

Reviewer #1: Yes

Reviewer #2: Yes

4. Is the manuscript presented in an intelligible fashion and written in standard English?

PLOS ONE does not copyedit accepted manuscripts, so the language in submitted articles must be clear, correct, and unambiguous. Any typographical or grammatical errors should be corrected at revision, so please note any specific errors here.

Reviewer #1: Yes

Reviewer #2: No

5. Review Comments to the Author

Please use the space provided to explain your answers to the questions above. You may also include additional comments for the author, including concerns about dual publication, research ethics, or publication ethics. (Please upload your review as an attachment if it exceeds 20,000 characters)

Reviewer #1: Important note: This review pertains only to ‘statistical aspects’ of the study and so ‘clinical aspects’ [like medical importance, relevance of the study, ‘clinical significance and implication(s)’ of the whole study, etc.] are to be evaluated [should be assessed] separately/independently. Further please note that any ‘statistical review’ is generally done under the assumption that (such) study specific methodological [as well as execution] issues are perfectly taken care of by the investigator(s). This review is not an exception to that and so does not cover clinical aspects {however, seldom comments are made only if those issues are intimately / scientifically related & intermingle with ‘statistical aspects’ of the study}. Agreed that ‘statistical methods’ are used as just tools here, however, they are vital part of methodology [and so should be given due importance].

COMMENTS: Your ABSTRACT is well drafted but assay type. Please note that it is preferable to divide the ABSTRACT with small sections like ‘Objective(s)’, ‘Methods’, ‘Results’, ‘Conclusions’, etc. which is an accepted practice of most of the good/standard journals [including this one]. It will definitely be more informative then, I guess, whatever the article type may be.

ANSWER: This is not according to the journals style (see link at the top of this document), but our abstract is actually following the organization proposed by reviewer 1.

REVIEWER 1: “Why [refer to ‘Availability of data’ section on page 14] the data analyzed in this article are added as Suppl. Mat.”?

ANSWER: It is a journal requirement that data are made available. An Excel sheet with all data sets are thus included so that anybody with access to appropriate software can repeat the analyses.

REVIEWER 1: Moreover, note that most of the ‘Correlations of AF, lipoproteins, physical activity and adiposity’ as reported/displayed in Suppl. Mat. 2 are generally ‘low’. In this context, please read the following [which is pasted from one standard textbook on ‘Research Methodology’]. I am sure that the authors already know these things, however, it is very essential to keep the limitations in mind while interpreting results {which unfortunately are not interpretated adequately}.
Statistical test usually used to assess significance of Pearson’s ‘Correlation coefficient (r)’ is ‘t’ [where t = { r √ [(n-2) / (1-r2)] }for df=n-2, n is sample size] and here Ho is that the population/standard value of ‘r’ is zero. You need r=0.878 to be significant at 5% when n=5 but you need r=0.273 only, if n=50 & you need r=0.088 only, if n=500. Because ‘P-value’ heavily depends on sample size, it is customary to use the (available in most text books on ‘Biostatistics’ or on ‘www/net’) guidelines [very strongly suggesting] to consider an absolute value of ‘Correlation coefficient’ for interpreting positive or negative correlations (and do not rely only on corresponding ‘P’-value but also consider an absolute value of ‘Correlation coefficient’). [This argument is equally applicable to non-parametric Spearman’s ‘Correlation coefficient (ρ)’ as well.]

ANSWER: The supplements with the Excel sheet with correlation coefficient (previously suppl. 2, now 3) and the patterns these correlations reveal (briefly discussed in suppl. 2, previously 1) were included to highlight the opposite patterns of physical activity and adiposity to aerobic fitness and the shared pattern of AF to high-intensity PA. This is given one sentence in the main manuscript simply to prepare the reader on the association patterns we can expect to find with the multivariate analyses. We do not test individually any of these correlations, only the *predictive* patterns are validated by doing real predictions using repeated Monte Carlo resampling which is a well-known and validated procedure for testing patterns for significance. If we would test the correlation for significance, we would have to follow the approach cited by the reviewer, but we would then also need to correct for multiple testing.

REVIWER 1: I request the authors to confirm the level of measurement of “Aerobic fitness test” scores [as said in concerned section that the test measured the total distance covered during a 10-minutes run. {and that Children ran from one end-line to another (20 m apart) in a to-and-fro movement intermittently, with 15-second work periods and 15-second breaks}. Moreover, you stated that “We used a PA descriptor of 23 intervals defined by counts per minute (cpm) covering the entire intensity spectrum”. In this context (just reminder), please read the following [which is again pasted from the same textbook on ‘Research Methodology’].
Though the measures/tools used are appropriate, most of them yield data that are in [at the most] ‘ordinal’ level of measurement [and not in ratio level of measurement for sure {as the score two times higher does not indicate presence of that parameter/phenomenon as double (for example, a Visual Analogue Scales VAS score or say ‘depression’ score)}]. Then application of suitable non-parametric test(s) is/are indicated/advisable [even if distribution may be ‘Gaussian’ (i.e. normal)]. Agreed that there is/are no non-parametric test(s)/technique(s) available to be used as alternative in all situation(s) [suitable / most desired/applicable], but should be used whenever/wherever they are available.

It is true that variable ‘time’ is ‘ratio’ but the procedure to record it could change level of measurement. One should just watch-out though it is definitely appreciable that “All variables were thus log-transformed, mean-centered and standardized to unit variance prior to the statistical analysis. After log transformation, normal probability plots showed that only CM, VLDL and a few of their subclasses in addition to TG still deviated from normal distribution”.

ANSWER: The data provided by accelerometry are provided in real time. The data on the sensor are preprocessed and summarized as minutes spent in defined intensity intervals over the time recorded. If one individual has twice as many counts as another individual in an intensity region, it implies twice the amount of physical activity in this region. So these physical activity variables are continuous and can be treated as such in analyses and tests, both univariate and multivariate.

We have added some crucial references that describe the sensor, preprocessing software and validation of the Andersen aerobic fitness test.

REVIEWER 1: Further I request the authors to read the article {just for information, authors are likely to have done this already} titled ‘Relative Importance of Borderline and Elevated Levels of Coronary Heart Disease Risk Factors’ (Ann Intern Med. 2005;142:393-402) & kindly note that I am not asking you to change the study design, but frankly speaking, ‘clinical aspects’ [like medical importance, relevance of the study, ‘clinical significance and implication(s) of the whole study, etc.] are not very clear and so in my considered opinion, should be assessed separately/independently.

ANSWER: The paper mentioned by the reviewer is an excellent paper using data for an adult population with participants from the well-known Framingham study and NHANES III. We agree that it would be nice if we could precisely assess the risk of adiposity and physical inactivity to cardiometabolic health as expressed by the lipoprotein association pattern in children and how this risk carry over to adulthood. To investigate the latter research question we would have to follow our cohort for decades. The former research question must consider the independent association of adiposity and physical activity, but also consider the inverse relationship of adiposity to physical activity. How is the lipoprotein association pattern independently associated to adiposity and physical activity and how is it associated with the joint influence of adiposity and physical activity? This is the research question we have tried to answer with our investigation, but the inverse relationship between adiposity and physical activity may underestimate the importance of physical activity if inactivity is the main causal reason for this relationship. To fully examine this relationship one need to do a study including measures of diet and genetic dispositions which are known to influence lipoproteins in addition to adiposity and physical activity.

Reviewer #2: The subject is of interest as these molecules may aid in pinpointing the molecular pathways linking health and disease in children and how they are influenced by lifestyle behaviours, such as physical activity, fitness and levels of adiposity. However, there are several issues described below that limit the interpretation of the findings generated

* I would consider renaming the title: Independent associations of aerobic fitness, physical activity and adiposity with lipoprotein subclasses in pre-pubertal children and also report the separate associations for PA and adiposity to the lipoprotein subclasses. This could be included as supplementary material. In my opinion, this is a more thorough approach with greater transparency.

ANSWER: In this work, we assess both the residual, the independent and joint influence of adiposity and physical activity on the association of aerobic fitness to the comprehensive lipoprotein profile. Our title was chosen to cover all these aspects. We have thoroughly examined the association of PA and adiposity to the lipoprotein patterns and published our findings in a separate paper. It was published just when we submitted the present paper. It is now included as the new reference 12 in the revised version of the paper. It is also mentioned in the discussion.

REVIEWER 2: * In the abstract, please include some basic information regarding your population. For example, the number of participants included, average age, BMI, waist circumference etc. It would also be helpful for the reader for this to be included in a Table in the main manuscript.

ANSWER: We have included a sentence describing the sample in the abstract. The Table asked for is already published in another paper studying the same cohort. We have given a precise reference to the appropriate Table in this publication in our revised paper. Furthermore, we have done the same for the physical activity variables.

REVIEWER 2: * The opening paragraph of the introduction contains one very long sentence. Consider revising.
ANSWER: The long sentence has been divided into three sentences.

REVIEWER 2: * Please make it clear in the second paragraph of the introduction that you are referring to your previous work. The way it is worded presently implies that you are referring to the current study
ANSWER: We understand this sentence was not clear and it has been rephrased.

REVIEWER 2: * The last two sentences of the introduction would be better placed in the discussion

ANSWER: We have rephrased slightly and incorporated the new reference 12 (our study of the association of adiposity and physical activity to the lipoproteins). We think it is important to mention the challenges our aims pose for the analysis in the introduction.

REVIWER 2: * More information is required regarding the accelerometers. For example, how did you define non-wear time? How many valid days were required as a minimum? It is also important to adjust for wear-time in the analysis. You also state that time spent in PA intensities obtained from the vertical axis was calculated for the subjects. Please include this information

ANSWER: This is now incorporated in the paper with some additional references as well. We do not adjust for wear time as previous investigations of this cohort showed such adjustment did not change our results. Probably due to the inclusion criteria we had for valid data, but also because the patterns are robust to differences in wear time. The information of PA intensities for the subjects are included on Excel sheets for all the data sets analyzed. Summary statistics for the PA descriptor of 23 variables is published in ref. 27.

REVIEWER 2: * As a minimum, you need to state that your analysis is observational, meaning that you cannot prove biological mechanisms or demonstrate causality; reverse causality is also a possibility whereby those with a greater burden of obesity may be less likely to engage in greater volumes or intensities of physical activity

ANSWER: It is now mentioned that the cross-sectional design is a limitation of the study.

REVIEWER 2: * Assuming this data is not available, the lack of adjustment for important confounders (i.e. diet quality, birthweight and socioeconomic status) needs to be addressed.

ANSWER: Diet is well-known to be important for lipoprotein levels so information on diet would clearly strengthen this study. We now have mentioned this as a limitation of the study.

Birthweight is not available in this study, but we have not seen any studies implicating that this should influence lipoprotein levels in children later in life. We have some measures of socioeconomic status in the ASK cohort, but this will only indirectly influence lipoprotein levels through lifestyle factors such as diet and physical activity which again influence adiposity. But we would not want to reduce the variation in adiposity or physical activity by adjustment for socioeconomic status even if we had a good descriptor for this. On the contrary, we want to embrace these variations in this work to not diminish the variation range of adiposity and physical activity. The rural cohort we have studied is also rather homogeneous with respect to socioeconomic factors. For a more heterogeneous cohort, a study linking socioeconomic status directly to lipoprotein levels might be interesting.

REVIEWER 2: * The resolution for Figure 1 is too low to review

ANSWER: It has been checked on the journal´s website with PACE and it appears to be a result of the pdf built on the website.

6. PLOS authors have the option to publish the peer review history of their article ([what does this mean?](https://journals.plos.org/plosone/s/editorial-and-peer-review-process#loc-peer-review-history)). If published, this will include your full peer review and any attached files.


If you choose “no”, your identity will remain anonymous but your review may still be made public.


**Do you want your identity to be public for this peer review?** For information about this choice, including consent withdrawal, please see our [Privacy Policy](https://www.plos.org/privacy-policy).

Reviewer #1: No

Reviewer #2: No

[NOTE: If reviewer comments were submitted as an attachment file, they will be attached to this email and accessible via the submission site. Please log into your account, locate the manuscript record, and check for the action link "View Attachments". If this link does not appear, there are no attachment files.]

While revising your submission, please upload your figure files to the Preflight Analysis and Conversion Engine (PACE) digital diagnostic tool, <https://pacev2.apexcovantage.com/>. PACE helps ensure that figures meet PLOS requirements. To use PACE, you must first register as a user. Registration is free. Then, login and navigate to the UPLOAD tab, where you will find detailed instructions on how to use the tool. If you encounter any issues or have any questions when using PACE, please email PLOS at [figures@plos.org](mailto:figures@plos.org). Please note that Supporting Information files do not need this step.
